# A molecular reconstruction approach to site-based 3D-RISM and comparison to GIST hydration thermodynamic maps in an enzyme active site

**DOI:** 10.1371/journal.pone.0219473

**Published:** 2019-07-10

**Authors:** Crystal Nguyen, Takeshi Yamazaki, Andriy Kovalenko, David A. Case, Michael K. Gilson, Tom Kurtzman, Tyler Luchko

**Affiliations:** 1 Skaggs School of Pharmacy and Pharmaceutical Sciences, University of California San Diego, La Jolla, California, United States of America; 2 Vancouver Prostate Centre, Vancouver, British Columbia, Canada; 3 National Institute for Nanotechnology, National Research Council of Canada, Edmonton, Alberta, Canada; 4 Department of Mechanical Engineering, University of Alberta, Edmonton, Alberta, Canada; 5 Department of Chemistry, Lehman College, The City University of New York, Bronx, New York, United States of America; 6 Department of Chemistry and Chemical Biology, Rutgers University, Piscataway, New Jersey, United States of America; 7 Department of Physics and Astronomy, California State University, Northridge, California, United States of America; Wake Forest University, UNITED STATES

## Abstract

Computed, high-resolution, spatial distributions of solvation energy and entropy can provide detailed information about the role of water in molecular recognition. While grid inhomogeneous solvation theory (GIST) provides rigorous, detailed thermodynamic information from explicit solvent molecular dynamics simulations, recent developments in the 3D reference interaction site model (3D-RISM) theory allow many of the same quantities to be calculated in a fraction of the time. However, 3D-RISM produces atomic-site, rather than molecular, density distributions, which are difficult to extract physical meaning from. To overcome this difficulty, we introduce a method to reconstruct molecular density distributions from atomic-site density distributions. Furthermore, we assess the quality of the resulting solvation thermodynamics density distributions by analyzing the binding site of coagulation Factor Xa with both GIST and 3D-RISM. We find good qualitative agreement between the methods for oxygen and hydrogen densities as well as direct solute-solvent energetic interactions. However, 3D-RISM predicts lower energetic and entropic penalties for moving water from the bulk to the binding site.

## Introduction

Water is intimately involved in the interactions of small molecule drugs with their protein targets. Implicit solvent models offer one approach to gain insight into the role of water in binding, but they do not capture all the molecular detail thought to be relevant in the confined, heterogeneous setting of a protein binding site. Explicit solvent models capture more detail, but do not directly provide insight into the role of water in a given binding site that can be used to guide ligand design. The last decade has seen a proliferation of computational tools and methods that address this gap by describing the hydration of protein binding sites and accounting in increasing detail for the role of water in the binding of small molecule drugs to their protein targets.

Two broad approaches, the Ornstein-Zernike (OZ) equation[1] and inhomogeneous fluid solvation theory (IST)[2–5], derive from the statistical mechanics of liquid state systems and have found application in academia and the pharmaceutical industry. Both OZ and IST are distribution function theories, in that they treat the fluid as a statistical distribution of particle or molecular densities. Although the formulation of each method is formally exact, computational implementation of these methods requires the use of approximations. The result is a mapping of local solvation thermodynamic properties, such as densities, energies and entropies.

In IST, explicit solvent Monte Carlo (MC) or molecular dynamics (MD) simulations are used to sample configurations from the Boltzmann distribution, and thermodynamic quantities are calculated from these samples. Although the molecular and atomic distribution functions generated from the simulations should asymptotically approach the correct results for the force field used, finite sampling means that IST results will have statistical uncertainties. Also, due to the limited sampling, the expression for entropy, which is represented in terms of single and multi-body molecular correlation functions in IST, must be truncated at low order (generally 1^st^, but occasionally 2^nd^ order[6–9]), leading to incomplete evaluation of the entropy. The IST approach has been implemented in at least two ways, one in which water properties are evaluated only at highly occupied hydration sites, as in WaterMap[10,11] and STOW[12]; and another in which water properties are evaluated for all voxels of a three-dimensional grid in the region of interest, as in GIST[13,14] and SSTMap[15].

The 3-dimensional reference interaction site model (3D-RISM)[16] is a widely used approximation to the molecular OZ equation. Like GIST, this approach also utilizes a simulation force field and water model, but it does not require sampling from the Boltzmann distribution. Instead, distribution functions for the system of interest are generated from an interaction-site approximation to the OZ equation, which is an exact relationship for the model system expressed in terms of the direct and indirect correlations between pairs of particles. As a consequence, 3D-RISM is much faster computationally than IST approaches, and it does not have statistically uncertainties due to finite sampling. On the other hand, as with all theories based on the OZ equation, solving the 3D-RISM equation requires a second equation, known as the closure relationship[17], which is necessarily approximate. In particular, the full closure relation contains an infinite series of many-body correction functions, and it must, in practice, be truncated at low order. As a consequence, the atomic distribution functions provided by the method are approximations. Exact expressions for the local thermodynamic properties consistent with the approximations are then applied to the distribution functions to yield the relevant entropies, energies, and densities. As further considered below, these quantities may be mapped to a three-dimensional spatial grid, much as done by GIST and SSTMAP in the IST approach (above). The error associated with these thermodynamic quantities traces to any error in the force field used, and to the approximations used to compute the atomic distributions. In recent years, corrections to errors in thermodynamic quantities have been developed that are applied after the solvent density distributions have been calculated, leading to estimates of solvation free energies with accuracies as good as explicit solvent simulations[18–29].

Given the similarities between grid-based IST implementations and 3D-RISM, it is clearly of interest to compare the two approaches. However, there has until now been a major obstacle to making such a comparison. Whereas GIST yields molecular distribution functions, 3D-RISM provides separate spatial distributions of the hydrogen and oxygen atoms of the water molecules, and of their contributions to the thermodynamics. When the oxygen and hydrogen contributions to the thermodynamic density distributions are additively combined, the resulting function has large variations in amplitude over very small distances. This causes problems when attempting to estimate the free energy of a ligand displacing water from a binding site into bulk; a slight shift in the position of a ligand may produce very different predictions of the energetic cost of displacing the solvent. This problem has been addressed by either averaging the contributions of solvent over coarser grids[30] or collapsing them on to solvation sites[31], but both approaches sacrifice the detailed information contained in the density distributions.

Here we report a new method to combine the oxygen and hydrogen contributions from 3D-RISM, based on the internal structure of water, into molecular density distributions and directly compare them to GIST results. Both methods are applied to the same conformation of the same protein, Factor Xa, using matching grid geometries and essentially the same force field. The Methods section provides a brief review of GIST and 3D-RISM, then explains the molecular reconstruction approach for 3D-RISM, as well as a method for mapping the partial molar volume correction (above) to the 3D-RISM grids. The Result section analyzes and compares GIST and 3D-RISM results for the spatial distributions of water molecules, energy, and entropy, within the binding site of Factor Xa. The Discussion session summarizes key points and considerations directions for future analysis of these two methods.

## Methods

### Grid inhomogeneous solvation theory

Grid inhomogeneous solvation theory (GIST), a grid based implementation of Inhomogeneous Fluid Solvation Theory, maps out thermodynamics densities on a high-resolution grid[13,14]. Software implementing GIST is available in the SSTMap[15] and AmberTools cpptraj[32,33] software packages. Inhomogeneous fluid solvation theory and the GIST grid implementation of it are detailed elsewhere, but we give a brief outline here for the reader’s convenience.

GIST utilizes system configurations sampled from the Boltzmann distribution, with molecular dynamics or Monte Carlo simulations, to compute spatial distributions of entropy and energy on a three-dimensional grid composed of cubic voxels. We focus here on the single-molecule (1^st^ order) entropy contributions and the 1^st^ and 2^nd^ order energy contributions. The equation for 1^st^ order approximation to the entropy is given by

$$S_{\text{sw}}=-\frac{k_{b}\rho^{0}}{8\pi^{2}}\int g\left(r,\omega \right)\text{ln}g\left(r,\omega \right)d\omega dr$$ (1)

where *k*_*b*_ is the gas constant, and *g*(**r**,*ω*) is the solute-water pair distribution function (PDF); i.e., the probability density of water at position **r** with an orientation of *ω*. Using the relationship:

g(r,ω)=g(ω|r)g(r) (2)

and the approximation that the orientational distribution is independent of the position within each cubic grid point, or voxel, the entropy of each voxel *k* with volume *V*_*k*_ can be separated into translational and orientational terms.

$$\Delta S_{\text{sw},k}^{\text{trans}}=-k_{b}\rho^{0}\int_{V_{k}}g\left(r\right)\text{ln}g\left(r\right)dr$$ (3)

$$\Delta S_{\text{sw},k}^{\text{orient}}=\rho^{0}\int_{V_{k}}g\left(r\right)S^{\omega }\left(r\right)dr$$ (4)

$$S^{\omega }\left(r\right)=\frac{-k_{b}}{8\pi^{2}}\int dr\int g\left(\omega |r\right)\text{ln}g\left(\omega |r\right)d\omega$$ (5)

Here *g*(**r**, *ω*) is the density of water at position **r**, *g*(**r**|*ω*) is the conditional density probability of finding a water with an orientation *ω* given a water at position **r**, and the subscript “sw” denotes the solute-solvent correlations. All the density probabilities in these equations are normalized by the bulk water density, *ρ*^*0*^, such as *g*(**r**) = *ρ*(**r**)/*ρ*^*0*^. In practice, the translational and orientational integrals may be calculated by histogram and/or nearest neighbor method[13,34,35].

The 1^st^ order (solute-solvent) and 2^nd^ order (solvent-solvent) energies are given by:

$$\Delta E_{\text{sw}}\left(r_{k}\right)=\int_{V_{k}}\Delta E_{\text{sw}}\left(r\right)dr$$ (6)

$$\Delta E_{\text{sw}}\left(r\right)=\frac{1}{8\pi^{2}}\int g_{\text{sw}}\left(\omega |r\right)U_{\text{sw}}\left(r,\omega \right)d\omega$$ (7)

$$\Delta E_{\text{ww}}\left(r_{k}\right)=\rho^{0}\int_{V_{k}}g_{\text{sw}}\left(r\right)\Delta E_{\text{ww}}\left(r\right)dr$$ (8)

$$\begin{array}{ll} \Delta E_{\text{ww}}\left(r\right) & ={\left(\frac{1}{8\pi^{2}}\right)}^{2}\rho^{0}\int g_{\text{sw}}\left(\omega |r\right)\\ & \left[g_{\text{sw}}\left(r^{\prime },\omega^{\prime }\right)g_{\text{ww}}\left(r,\omega ,r^{\prime },\omega^{\prime }\right)-g^{0}{}_{\text{ww}}\left(r,\omega ,r^{\prime },\omega^{\prime }\right)\right]\\ & U_{\text{ww}}\left(r,\omega ,r^{\prime },\omega^{\prime }\right)d\omega dr^{\prime }d\omega \end{array}$$ (9)

Here *g*_sw_(**r**) = *g*(**r)**, *g*_ww_(**r**,*ω*,**r**’,*ω*’) is the pairwise distribution function between two water molecules with coordinates (**r**,*ω*) and (**r**’,*ω*’) in the solute frame of reference, *g*^*0*^_ww_(**r**,*ω*,**r**’,*ω*’) is the corresponding distribution in bulk water, *U*_sw_(**r**,*ω*) is the solute-solvent interaction potential, and *U*_ww_(**r**,*ω*,**r**’,*ω*’) is the solvent-solvent potential.

The GIST software computes these energy and entropy terms for each voxel on the grid and produces data files for visualization and further analysis. When comparing GIST with 3D-RISM, it is important to recognize that GIST assigns properties (energy and entropy) to a voxel based on the water molecules whose oxygen atoms are located in it. Thus, for example, the energy of a voxel includes the interactions of the hydrogens of these water molecules even though the hydrogens are not located in the same voxel. In contrast, 3D-RISM by default assigns voxel energies on an atomic, rather than a molecular basis. Thus, for example, if a voxel contains a water oxygen but not the water’s hydrogens, only the oxygen’s interactions are assigned to it.

### 3D-RISM

Details of 3D-RISM[16,36–38], its implementation in AmberTools[39,40], and analytic temperature derivatives[41,42], in particular, three-dimensional generalization of the analytical derivatives[43] can be found elsewhere. Here we briefly summarize the 3D-RISM theory and the use of temperature derivatives to calculate solvation energy and entropy contributions to the total solvation free energy.

The 3D-RISM equation is

$$h_{\alpha }\left(r\right)=\sum_{\gamma }\int c_{\gamma }\left(r-r\prime \right)\chi_{\alpha \gamma }\left(r\prime \right)dr^{\prime }$$ (10)

where *h*_*α*_(**r**) is the 3D total correlation function (TCF) of solvent site *α* around the solute and *c*_*α*_(**r**) is the 3D direct correlation function (DCF).

$$\chi_{\alpha \gamma }\left(r\right)=\omega_{\alpha \gamma }\left(r\right)+\omega_{\alpha \gamma }\left(r\right)\rho_{\gamma }h_{\alpha \gamma }\left(r\right)$$ (11)

is the site-site bulk solvent susceptibility obtained from dielectrically consistent RISM (DRISM), *ρ*_*γ*_ is the site number-density,

$$\omega_{\alpha \gamma }\left(r\right)=\sum_{\lambda =1}^{M_{\gamma }}\delta_{\alpha \gamma }\delta \left(r\right)+\frac{\left(1-\delta_{\alpha \gamma }\right)\delta \left(r-L_{\alpha \lambda }\right)}{4\pi L_{\alpha \lambda }^{2}}$$ (12)

is the intramolecular correlation function, *M*_*γ*_ is the multiplicity of site *γ* (*M*_o_ = 1 and *M*_H_ = 2), *L*_*αγ*_ is the distance constraint between site *α* and the *λ* position of site *γ* of the rigid solvent molecule, and *δ*_*αγ*_ and *δ*(**r**) are the Kronecker and Dirac delta functions.

As *h*_*α*_(**r**) and *c*_*α*_(**r**) are unknown functions, a closure equation is required in order to obtain iterative solutions. We will make use of the partial series expansion of order-*n* (PSE-*n*)[44] approximate closure equation,

$$g_{\alpha }\left(r\right)=\{\begin{array}{cc} \text{exp}\left(t_{\alpha }^{*}\left(r\right)\right) & t_{\alpha }^{*}\left(r\right)<0\\ \sum_{i=0}^{n}\frac{{\left(t_{\alpha }^{*}\left(r\right)\right)}^{i}}{i!} & t_{\alpha }^{*}\left(r\right)\geq 0 \end{array}$$ (13)

With

$$t^{*}{}_{\alpha }\left(r\right)=-\beta u_{\alpha }\left(r\right)+h_{\alpha }\left(r\right)-c_{\alpha }\left(r\right).$$ (14)

Here *β* = *k*_*b*_*T*, where *T* is the temperature and *k*_*b*_ is Boltzmann’s constant, *u*_*α*_(**r**) is the potential energy function between solvent site *α* and the solute, and *g*_*α*_(**r**) = *h*_*α*_(**r**)+1 is the water-solute PDF. When *n* = 1 we obtain the Kovalenko-Hirata (KH) closure[37] and when *n* = ∞ the hypernetted-chain equation (HNC)[45] is recovered. For a given closure it is possible to obtain the excess chemical potential, or solvation free energy, of the solute. For PSE-*n* this is

$$\Delta \mu^{\text{PSE-}n}=\sum_{}\int \Delta \mu_{\gamma }^{\text{PSE-}n}{}_{\alpha }\left(r\right)dr$$ (15)

$$\Delta \mu^{\text{PSE-}n}{}_{\gamma }\left(r\right)=k_{b}T\rho_{\gamma }\frac{h_{\gamma }^{2}\left(r\right)}{2}-c_{\gamma }\left(r\right)-\frac{h_{\gamma }\left(r\right)c_{\gamma }\left(r\right)}{2}-\frac{{\left(t^{*}{}_{\gamma }\left(r\right)\right)}^{n+1}}{\left(n+1\right)!}\Theta \left(h_{\gamma }\left(r\right)\right)$$ (16)

where Θ is the Heaviside function and *Δμ*^PSE-*n*^_*γ*_(**r**) is the local excess chemical potential density. For any closure with a path independent expression for the solvation free energy[41], it is possible to decompose Eq (16) into energy, *E*, and entropy, *S*, contributions,

$$\Delta \mu \left(r\right)=\Delta E_{T,V}\left(r\right)-T\Delta S_{T,V}\left(r\right).$$ (17)

Omitting the ‘*T*,*V*’ notation and taking the isochoric temperature derivative, *δ*_*T*_≡*T*(∂/∂*T*)_*ρ*_, of the solvation free energy, we have for the PSE-*n* closure

$$\begin{array}{ll} -T\Delta S_{\gamma }^{\text{PSE-}n}\left(r\right) & =T\left(\frac{\partial \Delta \mu_{\gamma }^{\text{PSE-}n}\left(r\right)}{\partial }\right)=\delta \Delta \mu_{\gamma }^{\text{PSE-}n}\left(r\right)\\ & =\Delta \mu_{\gamma }^{\text{PSE-}n}\left(r\right)+k_{b}T\rho_{\gamma }\{h_{\gamma }\left(r\right)\delta_{T}h_{\gamma }\left(r\right)-\delta_{T}c_{\gamma }\left(r\right)\frac{{\left(t_{\gamma }^{*}\left(r\right)\right)}^{n}}{n!}\\ & -\frac{1}{2}\left[\left[\delta_{T}h_{\gamma }\left(r\right)\right]c_{\gamma }\left(r\right)+h_{\gamma }\left(r\right)\delta_{T}c_{\gamma }\left(r\right)\right]\\ & \;-\frac{{\left(t_{\gamma }^{*}\left(r\right)\right)}^{n}}{n!}\delta_{T}t_{\gamma }^{*}\left(r\right)\Theta \left(h_{\gamma }\left(r\right)\right)\} \end{array}$$ (18)

Independent of the closure, the solvation energy density is then

$$\Delta E_{\gamma }\left(r\right)=\Delta \mu_{\gamma }\left(r\right)-\delta_{T}\Delta \mu_{\gamma }\left(r\right).$$ (19)

As with GIST, the solvation energy can be broken down into solute-water and water-water contributions. The solute-water energy density is given by

$$\Delta E_{\text{sw},\gamma }\left(r\right)=\rho_{\gamma }g_{\gamma }\left(r\right)u_{\gamma }\left(r\right)$$ (20)

and the water-water energy density is then

$$\Delta E_{\text{ww},\gamma }\left(r\right)=\Delta E_{\gamma }\left(r\right)-\Delta E_{\text{sw},\gamma }\left(r\right).$$ (21)

However, since 3D-RISM does not explicitly consider the orientation of the water molecules, it is not possible to separate translational and orientational contributions.

### Molecular reconstruction

As noted above, in 3D-RISM, the contributions to the thermodynamic quantities from a single water molecule’s hydrogens and oxygen may fall into different grid voxels, whereas in GIST, all interactions of the water molecule are associated with the voxel occupied by the oxygen atom. We now describe a method of generating a molecular mapping of properties, similar to that of GIST, from the grids provided by 3D-RISM.

Consider an arbitrary thermodynamic quantity *A*(**r**), such as Δ*E*_sw_(**r**) or -*T*Δ*S*(**r**), composed of the sum of contributions from all solvent sites, e.g., *A*(**r**) = ∑_α_*A*_*α*_(**r**). A molecular distribution can be approximately reconstructed around a central site, *α*, e.g., oxygen, using the structural information in the intramolecular correlation function, Eq (12),

$$A\left(r\right)\approx A_{\alpha }\left(r\right)+g_{\alpha }\left(r\right)\sum_{\gamma \neq \alpha }\omega_{\alpha \gamma }\left(r\right)*A_{\gamma }\left(r\right).$$ (22)

Here, the convolution, *, between *ω*_*αγ*_(**r**) and *A*_*γ*_(**r**) has the effect of placing *A*_*γ*_(**r**) a distance *L*_*αγ*_ away in all directions. This is then weighted by the number density distribution of site *α*, *g*_*α*_(**r**). The final expression is the superposition of the thermodynamic quantity density distribution due to site *α* and that of the other sites mapped onto the number density distribution of *α*. While it is possible to select any solvent site as *α*, for water, it is natural to use oxygen; e.g.,

$$A\left(r\right)\approx A_{\text{O}}\left(r\right)+g_{\text{O}}\left(r\right)\left(\omega_{\text{OH}}\left(r\right)*A_{\text{H}}\left(r\right)\right).$$ (23)

Because this reconstruction uses only the local information contained in *ω*_*αγ*_(**r**) it does not properly include non-local contributions due to the DCF. For example, contributions from the excluded volume of the solute, where the DCF contributes most, are set to zero for the contributions from the *γ* sites (e.g., hydrogen) due to the weighting by the PDF. For consistency, contributions in the excluded volume due to the *α* site (e.g., oxygen) are also set to zero. Contributions to *A*(**r**) from the excluded volume region are therefore neglected, and contributes to errors in Δ*E*_*ww*_(**r**), Δ*E*(**r**) and *-T* Δ*S*(**r**) but not Δ*E*_*sw*_(**r**). However, we find that when these quantities are corrected (see below) the contribution from the excluded volume is small, making the approximation valid.

### Partial molar volume corrections

When integrated over all space, Eqs (16), (18), and (19) provide the excess chemical potential, energy, and entropy, respectively. However, these values have significant errors when compared to values from experiment. Several corrections have been developed to mitigate these issues for the excess chemical potential[18–21]. Recently, we demonstrated that several of these methods provide good agreement with excess energies and entropies from experiment as well[24].

While these corrections are known to work well for the integrated quantities of Eqs (17), (18) and (19), we wish to test if they also have an impact on the spatial distribution of these quantities. To test this, we apply the Universal Correction with temperature dependence (UCT)[24] to the thermodynamic density distributions,

$$\Delta E^{\text{UCT}}\left(r\right)=\left(\sum_{\gamma }\Delta E_{\gamma }^{\text{PSE-}n}\left(r\right)\right)-a\left(v\left(r\right)-\delta_{T}v\left(r\right)\right)-Ta_{1}v\left(r\right)+b_{0}$$ (24)

$$-T\Delta S^{\text{UCT}}\left(r\right)=\left(\sum_{\gamma }-T\Delta S_{\gamma }^{\text{PSE-}n}\left(r\right)\right)+a_{1}Tv\left(r\right)+a\delta_{T}v\left(r\right)+b_{1}T$$ (25)

where ‘RISM’ identifies the uncorrected quantities from Eqs (18) and (19),

$$v=k_{b}T\chi_{T}\left(1-\sum_{\gamma }\int \rho_{\gamma }c_{\gamma }\left(r\right)dr\right)$$ (26)

is the partial molar volume, and *χ*_*T*_ is the isothermal compressibility calculated from dielectrically consistent RISM (DRISM)[46]. Four parameters, *a*_*0*_, *a*_*1*_, *b*_*0*_, and *b*_*1*_, are fit to experiment are related by

$$\begin{array}{l} a=a_{1}T+a_{0}\\ b=b_{1}T+b_{0}. \end{array}$$ (27)

As will be discussed below we do not attempt to perform a molecular reconstruction on Eqs (24) and (25) because the corrections provide no observable benefit outside the excluded volume of the solute and the corrections make the contribution from the excluded volume close to zero.

### GIST analysis

The GIST calculations used a 20.5 x 20.5 x 22.5 Å grid composed of cubic voxels with side lengths 0.5 Angstroms, and centered in the binding site of the protein Factor Xa. GIST results were computed based on a 100 ns MD simulation of Factor Xa, as now detailed.

The crystal structure of Factor Xa (pdbid: 1FJS) was prepared with the ligand Z34 removed in AmberTools software tleap using ff99SB[47]. TIP3P[48] water molecules were added to form a solvent medium with a minimum distance of 10 Å from the protein surface to the edge of the water box. Four disulfide bonds were connected. All crystal ions (1 Ca^2+^ and 1 Cl^-^) were kept, resulting in a +2 net charge for the system. The simulation box had dimensions of 66.5 x 72.2 x 60.9 Å with a total of 29338 atoms and 8557 water molecules.

All molecular dynamics simulations were run in Amber14 using pmemd.cuda on a single GPU. Computer time was 52 hours for 100ns simulation with a time step of 2 fs. A 9 Å cutoff was applied to all non-bonded interactions while PME was used to account for long-range interactions. SHAKE was used to constrain all covalent bonds involving hydrogen atoms during the simulations. Cartesian restraint is employed throughout the simulation with a force constant of 100 kcal/mol/Å^2^.

The system was first relaxed by using two minimization procedures. Each was done with the steepest descent algorithm for the first 1500 time steps followed by the conjugate gradient method with a maximum of 2000 time steps. The first minimization was performed on the water alone by keeping all the protein’s atoms restrained. The second allowed the water and the protein’s heavy atoms to relax. The minimized system was heated gradually, at constant volume, with an increment of 50K for 20ps from zero to 300K. The temperature was regulated by the use of Langevin dynamics with a collision frequency of 2.0 ps^-1^. An equilibration simulation of 10 ns was run by keeping the temperature at 300 K and regulating the pressure at 1 atm, via isotropic position scaling with a pressure relaxation time of 0.5 ps. An additional NVT equilibration process keeping the temperature and volume regulated was conducted for 5 ns. An NVT production run of 100 ns was then carried out, while simulated configurations were stored every 1 ps. To study convergence of the GIST results, we performed four additional simulations of 100 ns starting from the same minimized configuration. The last simulation stored configurations every 0.2 ps.

### 3D-RISM analysis

3D-RISM calculations were carried out on the protein reference structure from the MD simulation with all water molecules removed. cTIP3P water[39] was used as a close analogue to the TIP3P water used in the MD simulations. cTIP3P differs by adding a Lennard-Jones potential energy term for the hydrogens, which is necessary for the application of RISM theory; it ensures that water hydrogens do not catastrophically overlap with neighboring water oxygens. Bulk properties of cTIP3P at 298 K with a dielectric of 78.497 and a density of 55.345 M were calculated for 3D-RISM using DRISM[46]. The DRISM solution was obtained on a grid of 16,384 grid points, with a spacing of 0.025 Å, to a target residual tolerance 10^−12^ using the modified direct inversion of the iterative subspace (MDIIS)[49] to accelerate convergence. 3D-RISM and DRISM calculations were carried out in AmberTools 16[39,50,51], with 3D-RISM modified to produce 3D solvation thermodynamics maps. The 3D-RISM solutions were computed on a grid with a 0.5 Å grid spacing and a minimum buffer of 24 Å between the solute and the outer edges of the grid. The *center = 3* option, which rounds the position of the grid points within the reference frame, was used to align the 3D-RISM and GIST grids. For visualization purposes, the 3D-RISM grid was truncated to match that used for the GIST analysis. The 3D-RISM equation was solved to a tolerance of 10^−6^ with the PSE-3 closure, using previous solutions obtained with the KH and PSE-2 closures, and accelerated by the (MDIIS) solver. Solute-solvent interaction potentials were calculated with an infinite cut off. The calculation took about 30 min on a quad core 3.5 GHz Haswell Intel Core i5 processor.

**Fig 1:**
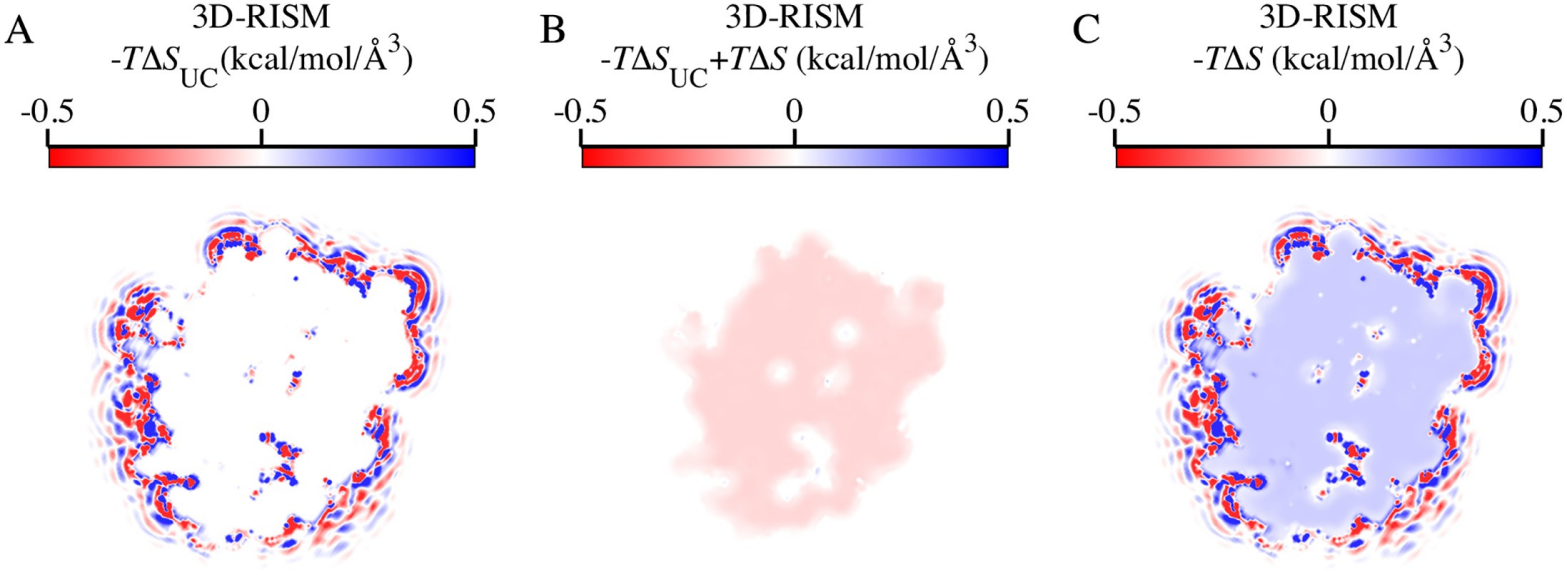
Cross section of the additively combined oxygen and hydrogen site solute-water energy density distributions about Factor Xa. Calculation from (A) 3D-RISM-PSE-3-UCT, (C) 3D-RISM-PSE-3, and (B) the difference.

To test the relevance of PMV corrections to thermodynamic density distributions, we applied the UCT with parameters of *a*_*1*_ = 0.0327564, *a*_*0*_ = -0.000507492, *b*_*1*_ = -3.26166, and *b*_*0*_ = 0.0100166, based on our unpublished results for cTIP3P using the same protocol as Ref.[24]. We have previously found for small molecules[24] that these corrections can provide solvation energies and entropies that agree well with experiment. In particular, the corrections primarily affect the solvation entropies and provided only a small improvement in solvation energies. A key feature of these corrections is that they are applied after the solvent density distribution has been calculated with 3D-RISM. They affect only the integrand of Eqs (15), (18) and (19), and do not modify the site density distributions *g*_*α*_(**r**) or *c*_*α*_(**r**). The corrected integrand for thermodynamic properties, such as solvation entropy (Fig 1), can still be visualized in 3D.

While these corrections provide large improvements to the solvation entropy, the region of space they affect is restricted to the excluded volume of the solute, as is shown by the difference (Fig 1B) between the corrected (Fig 1A) and uncorrected (Fig 1C) distributions. There is negligible impact on the distribution of solvation energy or entropy outside of this core region, which is the focus of this paper. Since the thermodynamic distributions in the binding pocket of the protein are not modified, we present uncorrected output from 3D-RISM-PSE-3 throughout Results.

## Results

This section presents a comparison of molecular and thermodynamic density distributions generated by 3D-RISM and MD/GIST. First, we compare the PDFs for both oxygen and hydrogen. Next, we illustrate the benefits of the molecular reconstruction for 3D-RISM in the context of the solute-water energy density distributions. Finally, we compare the two methods’ results for the water-water energy, total energy and total entropy density distributions.

It is worth emphasizing that well-converged GIST results are based on MD simulations that capture the true distribution functions for the choice of system and force field, while the faster but approximate 3D-RISM method approximates these distribution functions. As a consequence, the GIST results for number densities and energy densities may be viewed as a reference data against which 3D-RISM may be evaluated. However, the GIST expression for the entropy is generally truncated after the single-body first term (Eq (5); but see Lazaridis[2]), so entropic contributions due to water-water correlations are neglected. In contrast, although 3D-RISM uses approximate distribution functions, it computes the full entropy, including both single-body and multi-body contributions for these functions. Based on these considerations, neither GIST nor 3D-RISM should be regarded as a reference touchstone for the hydration entropies.

### Oxygen and hydrogen distributions

**Fig 2:**
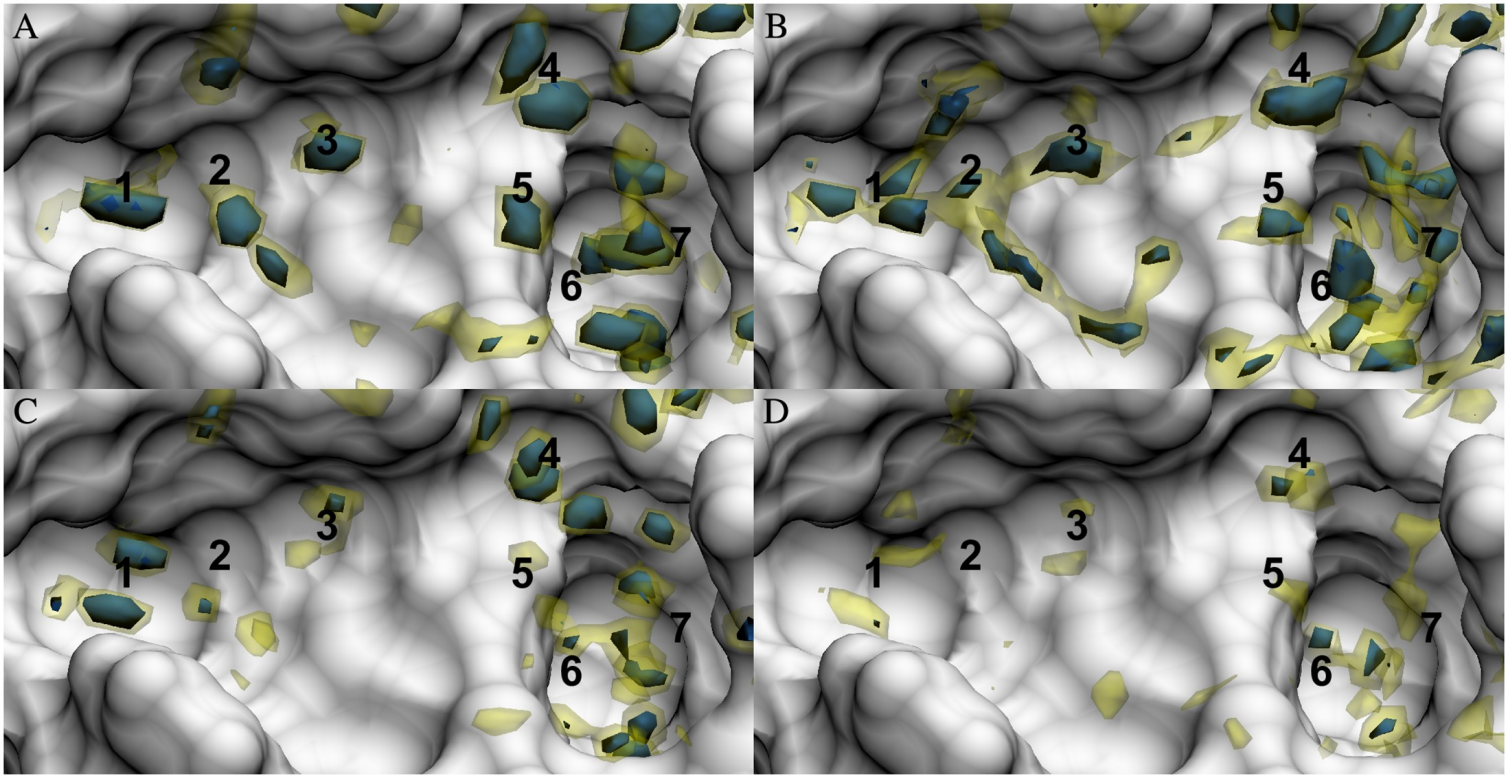
Solute-water pair distribution functions in the FXa binding site. Oxygen pair distribution functions as calculated by (A) MD and (B) 3D-RISM. Hydrogen pair distribution functions as calculated by (C) MD and (D) 3D-RISM. Isosurfaces are shown for 4 (yellow) and 8 (blue) times bulk density. The FXa solvent accessible surface is shown in white. Numbers identify density regions that are discussed in the text.

Number density distributions are the basic thermodynamic density distribution for GIST and 3D-RISM. Fig 2 shows the number density distributions relative to bulk generated by MD, which are used for GIST and 3D-RISM calculations. While there is overall agreement on high-density locations of oxygen and hydrogen, there are some cases where 3D-RISM and MD disagree on the position or existence of oxygen sites. In some cases, the position of a maximum is shifted. For example, the oxygen site found above location 4 in the MD distribution is moved up to the edge of the binding pocket in 3D-RISM. This is also the case, at location 7, where MD predicts a large oxygen density that is shifted much lower in the 3D-RISM distribution. Meanwhile, to the left of location 4, 3D-RISM predicts an oxygen site that is not observed in the MD distribution.

Overall, 3D-RISM has a less defined solvent distribution with lower, wider density maxima than found in the MD distributions. These results are consistent with previous comparisons of MD and 3D-RISM[52–54]. The difference can be clearly seen at locations 1, 2, and 3 in Fig 2. Here, MD presents highly localized oxygen sites while 3D-RISM generally has smaller high-density regions (8 times bulk density) but larger low-density regions (4 times bulk density). Low-density channels between the oxygen sites connect the high-density sites for both 3D-RISM and MD, but are only visible in MD at around 2 times bulk density (not shown).

For both MD and 3D-RISM, hydrogen distributions are less localized than the oxygen distributions with few peaks above 8 times bulk density. As with the oxygen distributions, MD has more density maxima than 3D-RISM and these have higher densities but there are no channels in either 3D-RISM or MD connecting the maxima. Both methods do locate some well-defined hydrogen sites, indicating highly oriented water molecules. At location 3 an oxygen site has a pair of well-defined hydrogen sites on either side, suggesting that this is the location of well-ordered and localized water molecule. Location 6 shows similar behavior deep within the binding pocket.

### Energy distributions

#### Solute-water energy density distributions

**Fig 3:**
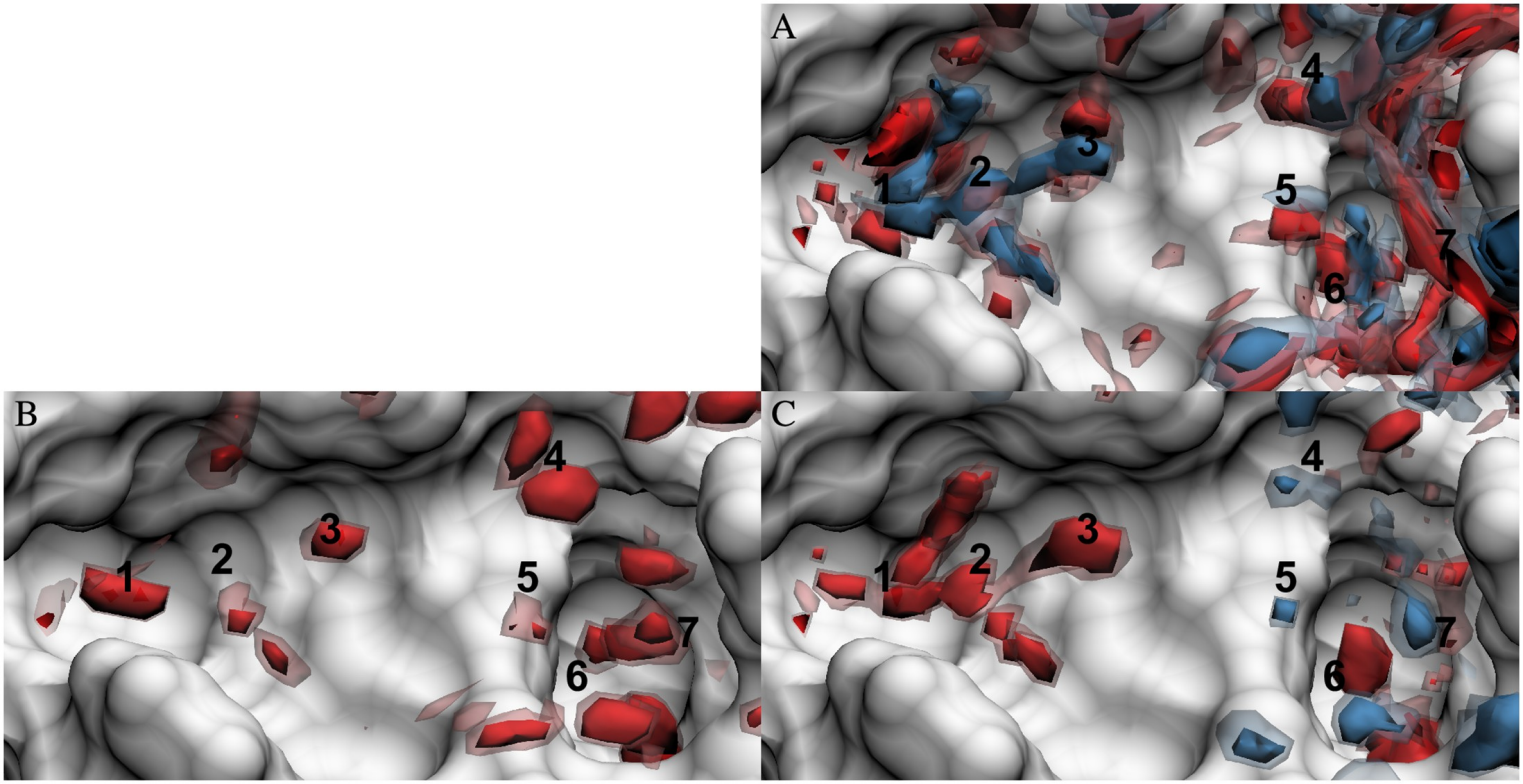
Solute-water energy density distributions in the FXa binding site. Energy density distribution were calculated using (A) 3D-RISM with additively combined oxygen and hydrogen site solute-water energy density distributions, (B) GIST, and (C) 3D-RISM with molecular reconstruction. Isosurfaces are shown for -2 (dark red), -1 (light red), 1 (light blue), and 2 (dark blue) kcal/mol/Å3. The FXa solvent accessible surface is shown in white. Numbers identify density regions that are discussed in the text.

Using Eqs (16), (18) and (19), it is possible to transform the oxygen and hydrogen site densities generated from 3D-RISM (Fig 2) into 3D density distributions of the solvation energy, entropy, and free energy. As noted in the Introduction and Methods, the site-site nature of 3D-RISM makes it difficult to interpret the direct visualization of thermodynamic density distributions. Fig 3A shows this for the solute-water energy density distribution, generated from Eq (20). On the right, we observe many positive and negative regions of the distribution in close proximity to each other. It is possible, when comparing to Fig 2, to identify contributions from hydrogen and oxygen separately. For example, at location 3, the contribution from the oxygen can be seen clearly in blue (positive energy) while the two localized hydrogen density maxima can be identified in red (negative energy). However, it is not possible to visually determine if a water molecule at this location would have positive or negative energy.

Applying Eqs (22) to (19) effectively combines the oxygen and hydrogen contributions and presents a molecular picture (Fig 3C), analogous to GIST (Fig 3B). Looking again at location 3, we see that the negative energy solute-hydrogen energy outweighs the positive solute-oxygen energy, leading to a favorable overall interaction between solute and water. This is the same result provided by GIST. Throughout the binding cavity, we see that the molecular reconstruction removes most of the unfavorable blue regions, leaving primarily favorable red regions. Because molecular thermodynamic densities are easier to interpret, we will only discuss the molecular reconstruction thermodynamic densities from this point on.

The resulting molecular solute-water energy density distribution from 3D-RISM qualitatively reproduces many of the features of the GIST density distribution. As with the oxygen density distributions, extrema from the two distributions significantly overlap. Both methods also show that the weakest energetic interactions are in the middle of the binding pocket, although there is still significant water density in this region.

There are several notable differences as well. Around locations 2 and 3, 3D-RISM solute-water energy density distributions are broader than those of GIST, though they still overlap well. The energy density peak from 3D-RISM at location 1 is also similar to GIST, except that the main peak is split in two. GIST also has a density above location 1, near the edge of the pocket, that is shifted down in 3D-RISM. Location 6 is similar except that 3D-RISM predicts a much broader minimum than GIST. In contrast, at locations 4 and 5, 3D-RISM and GIST disagree on the nature of the interaction: 3D-RISM predicts positive solute-water energies while GIST predicts negative energies. Pressure artifacts of the PSE-*n* closures are the most likely reason why 3D-RISM finds these locations to be energetically unfavorable, in contrast to the reference GIST result. The geometry of the binding cavity and the positions of neighboring water molecules will cause density maxima to form at these locations. Since there are no particularly strong electrostatic interactions between the solute and the water at these locations, the van der Waals forces will dominate. The lack of repulsive terms in the closures causes the solvent sites to be placed too close to the solute and the potential energy is then too high (see Discussion for details). While locations 4 and 5 clearly demonstrate this behavior, there are a few other locations in the binding pocket where similar disagreement occurs. Location 7 shows a different type of disagreement in that the significant negative solute-water energy found at this location is shifted down and is of much lower magnitude in the 3D-RISM distribution. This is consistent with the location of the oxygen density presented in Fig 2. While the water density is not well localized even in GIST, it seems to make a significant contribution to the solute-water energy.

#### Water-water energy density distributions

**Fig 4:**
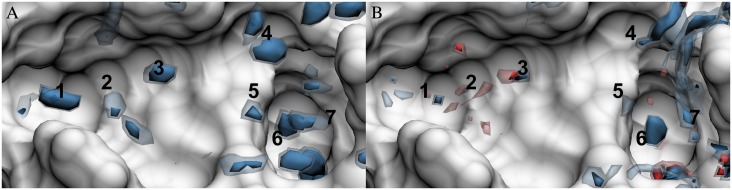
Water-water energy density distributions in the FXa binding site. (A) GIST and (B) 3D-RISM with molecular reconstruction. Coloring and numbering are as in Fig 3.

Water-water energy density distributions (Fig 4) indicate the change in energy of the current distribution relative to the bulk liquid, excluding interactions with the protein. These interactions should be unfavorable, in general, as water molecules in the binding pocket will not form the same hydrogen bond network as found in bulk water. This is the case for GIST, where all water-water energies are positive.

While 3D-RISM has points of agreement (locations 6 and 7) with the reference GIST results, 3D-RISM disagrees significantly with GIST in some regions, providing often neutral or favorable, rather than unfavorable, water-water interactions relative to bulk (locations 1–5). This is consistent with the overall impression that 3D-RISM water-water energies are more negative than those from GIST. A possible explanation for this is that bulk water from DRISM has less structure and fewer hydrogen bonds are broken when water moves from the bulk to the binding site. Thus, the loss of favorable interactions with other water molecules is less for 3D-RISM than it is for GIST. It is possible that the structure of the binding site even creates a template for improved water-water interactions within RISM theory. The lack of structure in DRISM bulk water is attributed to the well-known artifacts found in the PSE-*n* family of closures (see Discussion for details).

#### Total water energy density distributions

**Fig 5:**
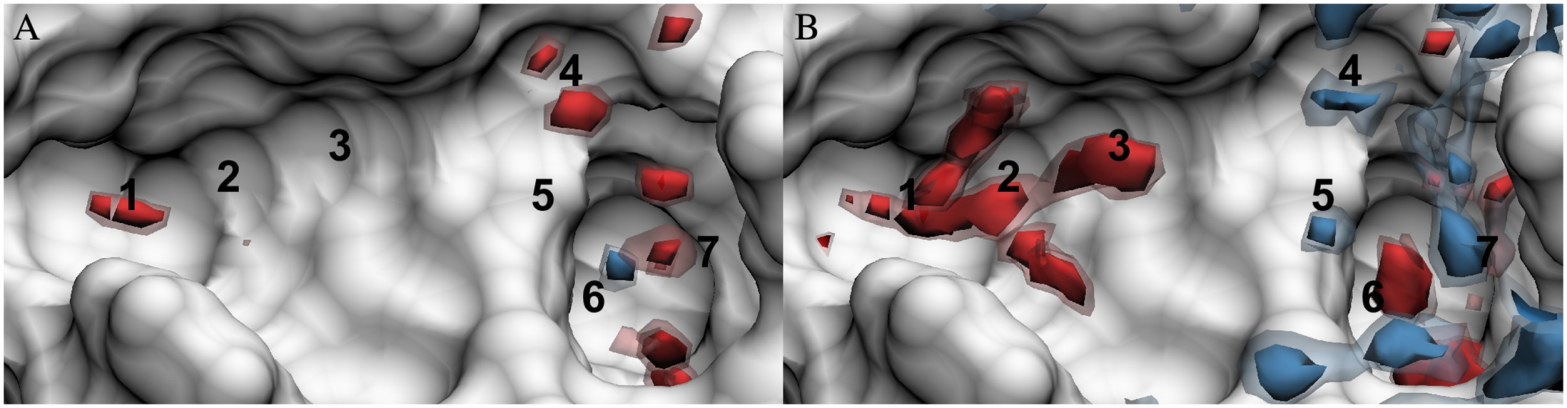
Total water energy density distributions in the FXa binding site. (A) GIST and (B) 3D-RISM with molecular reconstruction. Coloring and numbering are as in Fig 3.

When water-water and solute-water density distributions are combined to obtain total water energy density distributions, key differences already observed become magnified (Fig 5). The following analysis of specific locations illustrates the types of differences observed.

Locations 2 and 3 exemplify one type of deviation of 3D-RISM from the reference GIST results. Here, 3D-RISM reports a large area of favorable energies for the water, whereas GIST reports bulk-like energies. While both methods provide similar estimates of the solute-water energy, 3D-RISM underestimates the loss of the bulk water interactions, causing the total energy to be too negative. In contrast, at locations 4 and 5, 3D-RISM predicts positive total water energies whereas GIST predicts neutral or negative energies. While 3D-RISM again discounts the loss of water-water interactions, it also predicts repulsive interactions with the protein at these locations. Deep in the binding pocket, location 6, both GIST and 3D-RISM qualitatively agree on the density of oxygen with well-ordered hydrogens (Fig 2), the water-water energy (Fig 4), and the solute-water energy (Fig 3). However, the difference between the two energies results in a negative total energy from 3D-RISM while that of GIST is positive. Finally, at location 7, 3D-RISM does not assign a significant solvent density, and thus does not have the high energy density seen in GIST at this location.

### Entropy density distributions

As noted above, both GIST and 3D-RISM provide only approximate results for the entropy, and each makes different approximations. On one hand, GIST provides entropy under the assumption of only low-order correlations, and thus, cannot provide the full entropy, even in the limit of infinite simulation time. On the other hand, 3D-RISM accounts for all multi-body correlations, but its results are based on approximate distribution functions, due to the necessity of using an approximate closure relation. As a consequence, although it is useful to compare the two methods, neither can be relied upon as a reliable reference method for the other.

**Fig 6:**
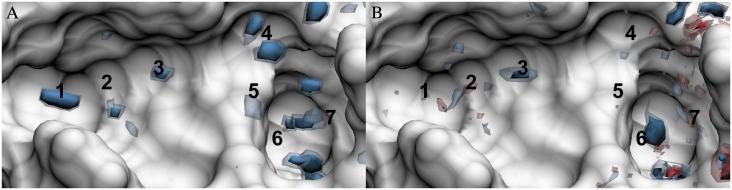
Total water entropy (-TΔS) density distributions in the FXa binding site. (A) GIST and (B) 3D-RISM with molecular reconstruction. Isosurfaces are shown for -2 (dark red), -1 (light red), 1 (light blue), and 2 (dark blue) kcal/mol/Å3. The FXa solvent accessible surface is shown in white. Numbers identify regions that are discussed in the text.

The density distribution of -*T*Δ*S* from GIST (Fig 6A) resembles the distribution of the water-water energy density from GIST (Fig 4A). In particular, both have peaks in similar locations, due largely to the fact that both thermodynamic densities are weighted by number density; and both are consistently unfavorable. Indeed, a consequence of the truncation of the entropy expansion to single-body interactions Eq (5) is that only unfavorable entropies, relative to bulk, are possible. It is thus worth noting that the neglected higher-order terms could, at least in principle, contribute enough to generate entropically favorable regions. For 3D-RISM, the entropy density distribution is smoother and, unlike GIST’s, includes some favorable regions (Fig 6B). Locations 3 and 6 are particularly well-peaked sites of unfavorable entropy. Overall, though, 3D-RISM has much lower entropy penalties for water to leave bulk and move to the surface of the protein.

## Discussion

### Overview

This study compares particle density and thermodynamic density distributions from 3D-RISM, analyzed with a new molecular reconstruction technique, and GIST, for essentially identical solutes, force fields, and grids. This is an excellent test of the number and energy density results from 3D-RISM, because GIST provides these distributions without approximation, except for the small numerical uncertainty associated with finite MD sampling in this case. It is also of interest to compare the entropy distributions, although it is important to note here that the GIST calculations omit the effects of water-water correlations, whereas these are included in 3D-RISM, so GIST cannot be viewed as a rigorous reference method for this term. Overall, we find good qualitative agreement between the number densities from the two methods, as they agree on the locations of most density peaks in the protein-water pair distribution function. However, 3D-RISM tends to produce less sharply peaked distributions than MD, which is a commonly observed difference for PSE-*n* and related closures[39,52–54]. The solute-water energy density distributions are also in general good agreement between the two methods, but the water-water energies deviate more. Indeed, GIST consistently shows only unfavorable contributions from this term, because water at the protein surface has fewer stabilizing interactions with other waters than does water in bulk; but 3D-RISM gives favorable water-water energies, relative to bulk at some locations. A similar picture emerges for the entropy terms, which, again, are consistently unfavorable in GIST, but occasionally favorable in 3D-RISM. As a consequence, 3D-RISM may tend to assign less thermodynamic benefit to displacing surface water from a binding site than does GIST.

### Accounting for the differences between GIST and 3D-RISM

A number of factors may contribute to the differences seen between GIST and 3D-RISM results. Naturally, dissimilarities in the density distributions produced by 3D-RISM and MD lead to differences between their thermodynamic distributions. 3D-RISM with the PSE-*n* family of closures is known to produce high density regions that are broader and lower peaked than those produced by MD[52,54]. These characteristics are observed in all of the density and thermodynamic distributions presented here. Small differences in the water models used and approximations in the molecular reconstruction procedure also contribute the differences observed between 3D-RISM and GIST results, though the amount is difficult to quantify.

Differences in the 3D-RISM density distributions from MD are partially due to well-known problems with the PSE-*n* family of closures. PSE-*n* closures are partial series expansions of the HNC closure. HNC (PSE-∞), in turn, approximates the full closure in terms of simple two-body correlation functions and misses the infinite series of many-body correlation functions contained in the full closure. These missing correlation functions are mainly repulsive, and their omission allows over-solvation, in which solvent sites form too close to each other or the solute[55], and results in seemly collapsed or distorted solvation structure.

When over-solvation occurs, hydrogen or oxygen sites approach the solute too closely and there is an increase in the magnitude of steric (positive) and electrostatic (negative) potential energies at the solute-water interface. Steric clashes can result in regions of positive solute-water energy density (Fig 3, location 4) while electrostatic interactions result in regions that are too negative (Fig 3, locations 1–3). This has previously been observed in other systems, such as 3D-RISM’s over representation of sodium ions around DNA phosphate backbones compared to explicit solvent molecular dynamics[54].

Over-solvation directly leads to global artifacts, such as excessively high pressure[53], which can be treated by PMV corrections. PMV corrections have been used successfully to mitigate the shortcomings of the PSE-*n* closures but we have found that these corrections have negligible impact outside the core region of the solute and are, therefore, unnecessary. This is due to the limited range of the direct correlation function, from which the PMV is calculated. As a result, these corrections only correct the *PV* work of introducing a solute into the solvent and do not significantly modify the details of the thermodynamics density distributions outside of the excluded volume of the solute.

The cTIP3P water model used with 3D-RISM, which is similar, but not identical, to the TIP3P model used in MD, also affects the structure of the solute-water pair distribution function. The most significant difference is the addition of a Lennard-Jones potential to the hydrogen sites, which is required to prevent catastrophic overlaps of hydrogen and water in RISM theory. In cTIP3P the Lennard-Jones diameters of hydrogen, *σ*_H_, is determined by *σ*_H_ = *σ*_O_-2*L*_OH_ = 1.2363 Å and enters 3D-RISM theory both through *χ*_*αγ*_(**r**) in Eq (10) and *u*_*α*_(**r**) in Eq (13). Unlike smaller values sometimes used with three-point water models, it gives the appropriate contact distance for hydrogen bonding. This greatly improves radial distribution functions of bulk water for OH and HH, particularly in improving the position and height of the first peak, but slight broadening of peaks in the OO distribution. cTIP3P, cSPC/E, and similar models have been widely used for the calculation of bulk water hydration thermodynamics with much success[18–21,26,39,42,53,56]. It is also worth noting that, through DRISM, we impose a dielectric constant of 78.497 on the water model to match experiment, compared to a value of 94 for TIP3P[57]. We expect this water model to give the best agreement with experiment when used with 3D-RISM; but it is not optimized to agree with explicit solvent MD.

Another factor that contributes to the differences found between GIST and 3D-RISM is the molecular reconstruction method we have developed. The molecular reconstruction remaps the hydrogen contributions to the thermodynamic density distributions of the parent oxygen using only intramolecular bond distances. This works well for *h*_*α*_(**r**) and the protein-water energy density as a result, which are local quantities. However, the water-water energy, Eq (21), and the total entropy, Eq (18), depend on non-local contributions from *c*_*α*_(**r**), *h*_*α*_(**r**) *c*_*α*_(**r**), and their temperature derivatives. While *c*_*α*_(**r**) contributes mainly to the core region of the protein, *h*_*α*_(**r**) *c*_*α*_(**r**) can influence the first solvation shell. Alternate reconstructions, that better account for *h*_*α*_(**r**) *c*_*α*_(**r**), may work better.

### Implications and directions

Both 3D-RISM and GIST provide detailed thermodynamic density distributions of solvent in binding cavities that can be used to guide the development of new pharmaceutical compounds. For systems like Factor Xa, where relevant water configurations can be easily sampled due to the solvent-exposed character of the binding site, GIST provides rigorous results at modest computational cost. Indeed, beyond the use of the force field, the only approximations in GIST are those due to sampling and truncation of the entropy calculation. On the other hand, exhaustive sampling is not possible in all systems. For example, in some systems water molecules may have frustrated dynamics or exchange with buried solvent pockets. In addition, if one wishes to examine solvation for multiple receptor conformations, the cost of running multiple MD simulations for GIST may become prohibitive. In such cases, the low computational cost of 3D-RISM can be of great benefit despite the approximations inherent in the theory. In future work, we will evaluate the potential benefits of both methods in the context of drug design.
